# Give Me What I Want: Identifying the Support Needs of College Student Entrepreneurs

**DOI:** 10.3389/fpsyg.2020.01428

**Published:** 2020-07-28

**Authors:** Peng Wang, Yangjie Huang

**Affiliations:** Institute of China Innovation and Entrepreneurship Education, Wenzhou Medical University, Wenzhou, China

**Keywords:** college student entrepreneurs, support needs, financial support, entrepreneurship education, psychological counseling

## Abstract

To determine how the success rate of college students’ entrepreneurship can be improved, we sought to identify the support needs of undergraduate entrepreneurs and evaluate the importance of different support needs using data from face-to-face interviews with 138 Chinese college student entrepreneurs. Further, we explored the distribution tendencies of the support needs at different entrepreneurial stages. We found that the support needs of college student entrepreneurs can be divided into 20 types, which can be categorized into core support needs (six types), secondary support needs (six types), and marginal support needs (eight types) in descending order of importance. A cross-stage analysis indicates that the support needs differ during the different entrepreneurial stages. While the needs during the entrepreneurial preparation stage and the startup stage have many similarities, those during the entrepreneurial failure stage are significantly different from those of the other three stages examined, showing heterogeneity. In addition, we identified psychological counseling as an important core support need in the failure stage and demonstrate its critical role in helping college students out of the shadow of entrepreneurial failure. This study expands the research on entrepreneurial support needs. Its findings provide valuable information to help colleges and governments develop more targeted and appropriate support for college entrepreneurs based on entrepreneurial stages to promote entrepreneurial success.

## Introduction

The landmark literature published in the early decades of the 20th century established entrepreneurship as a multidisciplinary research field spanning management, economics, psychology, and sociology, with each discipline’s contributions ceaselessly expanded and deepened by numerous scholars (Davidsson and Wiklund, 2001; Busenitz et al., 2003; Ireland et al., 2005; Dong et al., 2019; Wu and Song, 2019). The abundance of excellent entrepreneurial research has provided practical guidance for entrepreneurship (Grégoire et al., 2010). Entrepreneurship refers to the process by which people invest in, design, launch, and operate new businesses to sell ideas, products, or services to create a profit. Entrepreneurs have a need to create or identify opportunities and develop business ventures (Carland et al., 1984). Studies show that not only can entrepreneurship improve a grim employment situation and promote economic and social development, but can also serve as an important way to transform technologies into products (Grégoire et al., 2010; Premand et al., 2012). Given all this, entrepreneurship has received substantial support from governments, international organizations, universities, and other institutions.

Considerable entrepreneurship research over the past 50 years has focused on universities and academic entrepreneurship (Klofsten and Jones-Evans, 2000), university technology transfer (Siegel and Phan, 2005), and entrepreneurial learning and entrepreneurship education (Wee, 2004). This has provided a solid foundation for understanding the role of universities in students’ and graduates’ entrepreneurial activities. Despite this scholarly interest, not much is known about the role of college students in entrepreneurship. According to relevant survey results, the entrepreneurship rate among college students is often higher than that among professors, and students are more likely to engage in entrepreneurial activities (Fini et al., 2016). Research from the Massachusetts Institute of Technology suggests that all the companies founded by its alumni of both undergraduate and graduate could be worth as much as the GDP of world’s 10th-largest economy as of 2014 (Roberts et al., 2015). A survey in Italy found that 3% of Polytechnic University of Milan alumni create enterprises in the period between the year of enrollment in the second cycle degree and 5 years after graduation, and similar findings have been confirmed by other universities (Colombo et al., 2015; Fini et al., 2016). In addition, some studies have found that the support provided by universities is crucial to the success of both students and graduates who are starting businesses (Morris et al., 2013). Few enterprises achieve initial success without external support, including from colleges (Schleinkofer and Schmude, 2013).

In view of the above, it is of great theoretical and practical value to pay attention to the entrepreneurial activities of college students. The practical and research results have shown that, compared with mature entrepreneurs, college student entrepreneurs often have insufficient resources and capacity reserves. Consequently, their entrepreneurial success rate is low (Van Weele et al., 2016). This suggests that college student entrepreneurs need more support to prepare them for dealing with problems they might encounter in the process of entrepreneurship. Reasonable support for college entrepreneurs that meets their needs will undoubtedly improve their odds of success. Studies have used support needs as a collective term that includes entrepreneurship knowledge, management skills, product development, office space, access to investors, and other resources (Vandor et al., 2012; Van Weele et al., 2016). On this basis, some colleges offer entrepreneurship education, specialized training, and financial or physical support (Hindle and Cutting, 2002; Brown and Hanlon, 2014) to help entrepreneurial college students and graduates overcome obstacles to starting their own businesses. However, it is still worth discussing what level of entrepreneurship education, training, and other resources is most valuable for college entrepreneurs. Although many studies have indicated that the support needs of entrepreneurs are heterogeneous, varying with the entrepreneurs and the entrepreneurial stages, there has been little discussion on how this relates to college student entrepreneurs (Vandor et al., 2012). To fill this gap, this study addressed the following three research questions:

(1) What are the support needs of college entrepreneurs?
(2) Which support needs for college entrepreneurs are more important?
(3) How do the support needs for college entrepreneurs change during the process of starting a business?

To answer these three questions, we conducted face-to-face interviews to identify the various support needs of college student entrepreneurs, evaluated the relative importance of the needs, and detected the distribution tendencies of support needs during the different entrepreneurial stages. The findings will deepen the understanding of the support needs of college student entrepreneurs. Evaluating the relative importance of support needs can help colleges and governments provide appropriate support for college entrepreneurs to promote entrepreneurial success.

The remainder of this article is structured as follows: section 2 reviews the literature on entrepreneurial support needs and the entrepreneurial process; section 3 describes the research methods; section 4 presents the research results; section 5 provides the research discussion; and section 6 outlines the research conclusion.

## Literature Review

### Entrepreneurial Support Needs

The resource-based view regards an enterprise as an organization composed of various resources (Barney et al., 2001). To be successful, startups must constantly absorb external resources and improve the efficiency of internal resources. Research has shown that startups often face more resource constraints than established enterprises (Van Weele et al., 2016). In entrepreneurship, entrepreneurs need to integrate various resources to promote the development of startups, and it is useful to identify entrepreneurs’ support needs. Differences in research focus have identified and classified different support needs for startups. Some studies have focused on the impact of single support needs and explored the roles of providers of single and multiple forms of support (Pittaway and Cope, 2007). Other research has focused on this diversity in support needs (Vilanova, 2010). Barney (1991) divided firm resources into physical capital resources, human capital resources, and organizational resources; he concluded that the most critical support needs were in the area of human capital resources. Stinchecombe (1965) pointed out that the support needs of startups usually include relationships with suppliers and customers or established roles and routines within the firm. Timmons et al. (2007) divided support needs into people, financial resource, assets, and the business plan. Van Weele et al. (2016) systematically classified the support needs of startups into physical capital, financial capital, knowledge, social capital, and legitimacy. Some scholars have discussed the relationship between multiple support needs and multiple providers (e.g., Rydehell et al., 2018). In general, it is believed that entrepreneurs at different entrepreneurial stages have different entrepreneurial support needs, and several researchers focusing on time as a dynamic variable have pointed out that entrepreneurs’ support needs change with the different entrepreneurial stages (Vandor et al., 2012). For instance, in the startup stage, support is critical for propping up entrepreneurial opportunity discovery and entrepreneurial opportunity exploitation (Companys and McMullen, 2007; Ge et al., 2016). As a new enterprise grows and resources become more abundant, entrepreneurial teams change their decision goal to improve the efficient use of resources, and the support needs change with entrepreneurial stages. Thus, in the next section, we discuss the entrepreneurial stages and process reviewed in the published articles.

### Entrepreneurial Process

Many researchers (Singh et al., 1999; Anderson, 2000) have investigated the entrepreneurial process. Early research on the entrepreneurial process described it as the process of creating a new organization and discussed it from the perspective of entrepreneurial activities (Gartner, 1985). Scholars gradually realized that the entrepreneurship process should not be limited to the creation of a single enterprise, and began to investigate the entrepreneurship process more deeply. For example, Frese and Gielnik (2014) noted that the entrepreneurial process includes not only the creation of an enterprise but also its growth and development periods. Shane and Venkataraman (2000) argued that entrepreneurship is not one process but a series of processes involving opportunity identification and exploitation.

The literature primarily focuses on the entrepreneurial process from three perspectives. The first perspective emphasizes the elements of the entrepreneurial process and their interaction, often highlighting the personality characteristics particular to entrepreneurs. For example, Gartner (1985) broke the entrepreneurial process down into four elements—entrepreneur, entrepreneurial environment, entrepreneurial organization, and founding process—and emphasized the relationship among the four.

The second perspective of entrepreneurial process research focuses on the dynamics of the entrepreneurial process and usually introduces time into the mix. From this perspective, researchers have discussed the chronological sequence of entrepreneurial activities based on the enterprise life-cycle theory. For example, focusing on the growth process of a new enterprise, Galbraith (1982) divided the startup process into four stages: proof of prototype principle, model shop, startup and natural growth. Holt (1992) divided the entrepreneurial process into four stages: pre-startup, startup, early growth, and later growth. From the perspective of entrepreneur career development, some researchers have suggested other stages such as deciding to be an entrepreneur, selecting entrepreneurial opportunities, conducting preliminary analysis, building a management team, formulating a business plan, formulating an action plan, early operations and growth, and achieving personal and corporate success.

The third research perspective of the entrepreneurial process combines the first two research perspectives and proposes entrepreneurial process models that focus on both the entrepreneurial process and time dynamics. For example, Timmons et al. (2007) stated that the entrepreneurial process is a highly dynamic balance of entrepreneurial opportunities, entrepreneurial teams, and appropriate allocation of resources. Among these, he argued that the most critical were entrepreneurial opportunities, entrepreneurial resources, and entrepreneurial teams. Entrepreneurial opportunity is the core element of the entrepreneurial process (Timmons et al., 2007). In essence, the entrepreneurial process is an interaction process between entrepreneurs and new enterprises in an entrepreneurial external environment. In consideration of the characteristics of college students’ entrepreneurship and drawing on the entrepreneurial stage division method from Holt (1992), we divided the entrepreneurial process of college students into four stages: preparation, startup, maturity, and failure. We chose this four-stage entrepreneurial process model because compared with general entrepreneurs, college students’ entrepreneurship often lasts for a shorter duration and stalls in the initial period, and this analysis model reflects this difference. Considering the widespread existence of entrepreneurial failure, we also added that stage to our model. Finally, we adopted a four-stage model of an entrepreneurial process for the subsequent analysis. In the next section, we discuss the support needs of college student entrepreneurs at different stages.

### Support Needs of College Student Entrepreneurs

Studies have explored the support needs of entrepreneurs in general (Van Weele et al., 2016), female entrepreneurs specifically (Lockyer and George, 2012), and social entrepreneurs (Vandor et al., 2012), but there has been scant systematic research on the specific support needs of college student entrepreneurs. At present, most of the research on the support needs of college students has paid attention to financial support (Morris et al., 2017), entrepreneurship education (Hindle and Cutting, 2002; Brown and Hanlon, 2014), or some other forms of support, with a lack of systematic research on multiple support needs. Therefore, to fill this gap, this study focused on college student entrepreneurs and systematically explored the main types of support needs. Additionally, we evaluated the importance of different types of support needs to deepen our understanding of the support needs of college students. Our literature review of the entrepreneurial process revealed that entrepreneurs have different entrepreneurial support needs at different stages of the entrepreneurial process (Shane and Venkataraman, 2000; Bruyat and Julien, 2001; Timmons et al., 2007). For our study, we posited that college student entrepreneurs follow the same pattern, but we could not find supporting evidence in the literature. Therefore, we designed our study to investigate both this pattern and the distribution tendencies of support needs across the different entrepreneurial stages.

## Materials and Methods

### Research Design

Since the literature review showed that relatively few studies have examined the support needs of college student entrepreneurs, we determined that it would be best to adopt a qualitative method for our study. One way in which the qualitative research method differs from the quantitative research method is that the former involves the participation of researchers. In our qualitative study, we used an inductive approach to identify the support needs and evaluate their importance based on the theoretical paradigms of postmodernist philosophy. To achieve the purpose of this study, we selected a multi-case study method (Makhlouf and Allal-Chérif, 2019). The multi-case study method gives researchers a deeper understanding of the research subjects, enabling them to explore and discover new research problems and construct new research theories. The first step in a multi-case study is to choose the right cases. As a general rule, there are three typical sampling methods: theoretical sampling, objective sampling, and selective sampling (Sandelowski, 1995; Robinson, 2014). Since theoretical sampling is used for studies intended to develop theories, we chose this method for the current study.

This study selected cases that met three conditions: (1) the entrepreneurs were still college students; (2) the entrepreneurs were preparing to start businesses, had begun to engage in entrepreneurial activities, or had engaged in but failed at entrepreneurship; and (3) the entrepreneurs were willing to participate in this survey and showed a good attitude of cooperation.

### Data Collection

We mainly used face-to-face interviews to collect our qualitative data. Our research team interviewed 93 subjects. The subjects recruited for this study were all Chinese college students but were from different colleges in China. The investigators conducted interviews at their own colleges. We conducted interview training for the investigators through the Internet and by telephone, establishing guidelines for the open interviews (Table 1).

**TABLE 1:** Outline for open interviews.

Interview theme	Main content
Basic information of respondents	Please briefly introduce yourself and your entrepreneurial team.
Entrepreneurial practice or entrepreneurial experience	Please tell us about the entrepreneurial practice or personal entrepreneurial experience you have participated in.
Entrepreneurial process and stage	Which entrepreneurial cycle is your current entrepreneurial team in? (i.e., preparation, startup, maturity, or entrepreneurial failure).
Support needs	What kind of support do you need most at this stage? Why?

To ensure quality of the interviews, we required the investigators to conduct them based on our outline. Before each interview, the investigators were required to communicate the interview content to the interviewees by telephone or email (see Table 1). The actual interviews were carried out according to the outline but were not limited to the outline. The interview sites were generally selected by the interviewees and were usually quiet, independent offices or private areas in coffee shops. The interviews ranged from 30 to 60 min. In addition, during the interview, the investigators not only electronically recorded the interview information but also observed (and later noted on paper or electronic notes) the facial expressions, body movements, and tone of the interviewees to assist in subsequent analysis.

After each interview was completed, we asked the investigator to organize the interview content into a Microsoft Word file and submit it to our research team. Some interview records were found to be incomplete and returned to the interviewers; once the missing interview data were added, we included the completed interviews in our analyses. After the interview, we paid each researcher according to the actual research situation. Following a preliminary analysis of the data, we added some interview data from 45 college entrepreneurial students using the same data collection method. At this point, the data reached saturation, and we stopped the collection of research data. It should be noted that to effectively implement triangular verification of the data, the study also collected some second-hand data and materials, consisting mainly of (1) relevant information about entrepreneurs and entrepreneurial teams that was displayed by the colleges; (2) promotional materials or information about entrepreneurs or entrepreneurial teams directly collected from interviewees; and (3) relevant information we found online, including but not limited to company introductions, product introductions and main businesses. We compared these independent data from different sources with the interview data we collected for our data analysis to find consistencies and differences. In this way, we were able to realize the mutual confirmation of data from different sources, complement the data, and ensure the validity of data analysis (Yan and Gray, 1994).

We interviewed 138 college student entrepreneurs from 104 college student entrepreneurial teams. The students’ ages ranged from 19 to 24, and there were 120 male and 18 female subjects. The college students’ entrepreneurial teams were involved in information technology, e-commerce, biomedicine, media, energy, traditional commerce, and some other industries.

### Research Data Analysis

To answer the main research questions, this study extracted research topics from a large volume of qualitative data. For this purpose, we adopted the coding method to analyze our data. Specifically, we coded and analyzed the primary data and used the second-hand data as verification evidence to enhance the validity of the research conclusions. At present, there are two main coding methods: completely open coding and coding according to theory. Mindful that relatively few theoretical studies on the support needs of college student entrepreneurs have been conducted, we adopted the first coding method for our study (Corbin and Strauss, 1990). In the process of coding, we followed the principle of live coding, which extracts as many of the interviewees’ original words as possible. This coding method can help avoid introducing personal biases during data analysis. In the coding process, the two coders coded independently, and we achieved the final determination of coding by mutual confirmation. For the disputed coding, we recruited a five-person consultation group to help us make the final coding determinations for the study; this also helped reduce research bias in the study. In this process, we also asked ourselves the following questions to verify and correct the coding:

What is the relationship between these materials and the research?
What concepts have these materials produced, and what are the similarities and differences among these concepts?
What are the areas of entrepreneurship research that can be identified to summarize these concepts?

This helped reduce coding bias as much as possible. Finally, after a group discussion, we identified 450 live codes and conceptualized them into 20 confirmed categories representing the most common support needs of college student entrepreneurs.

After completing the original qualitative data coding, to answer the second research question, we used the frequency statistics method to analyze the frequency of support needs of college student entrepreneurs (Parker-Rhodes and Joyce, 1956). The frequency analysis method is commonly used to evaluate the importance of research objects. Generally, the more frequently an object appears, the more important it is. Thus, in this study, the more times a certain type of support need was mentioned in the sample, the more important it was. Then, according to the percentage of single support needs to total support needs, we categorized the support needs into core support needs (>5%), secondary support needs (>2%), and marginal support needs (<2%).

To answer the third research question, we conducted additional comparative statistical analyses of the distribution of entrepreneurship support needs among college students during the preparation, startup, maturity, and failure stages to detect the distribution tendencies of entrepreneurial support needs across the process.

### Research Reliability and Validity

Qualitative research does not pay as much attention as quantitative research to the reliability and validity of the data. However, we still tried to ensure the reliability and validity of our research findings (Yin, 2003). For example, we adopted a triangulation strategy for the different sources of data to ensure the validity of the study during the data collection period (Yan and Gray, 1994). In the process of data analysis, we ensured the reliability and validity of the study by making detailed interview plans, writing memos, using independent coding by multiple coders, and engaging in group discussions. In addition, we confirmed that the support needs identified in our research had appeared in other researchers’ studies, which supported our study’s reliability and validity.

## Results

### Analysis of Support Needs of College Student Entrepreneurs

The support needs of college student entrepreneurs are shown in Table 2. Through the coding of the interview data, we identified 20 types of support needs. We describe each support need separately.

**TABLE 2:** Process and results of open coding.

Representative quotation	Codes
At this stage, what we need is entrepreneurship competitions. We aspire to be able to speak about our ideas in a competition. (Case 29) I hope to participate in a business planning competition as soon as possible so that I can judge whether my idea is feasible and determine my next action plan. (Case 20) We are encouraged to take part in competitions to test our ideas, and it’s very useful for me. Besides, maybe I can get some rewards. (Case 8)	Entrepreneurship competitions

Because the business of our company is not very stable, if the rent of our office space is added, it will have a great impact on our company. (Case 17) At present, what we need most is not only the assistance of professional mentors from the entrepreneurship college but also an office space suitable for our team. (Case 45) We are now in the startup stage, and we hope that the school can provide us with office space and teacher guidance. (Case 30)	Office space

What we need most is the sharing of entrepreneurial information. (Case 42) Schools should share more business success stories. (Case 54) However, I think the atmosphere at the beginning is very important, and it has a great influence on people’s ideological understanding in the startup stage. (Case 12)	Entrepreneurial climate

We need help on how to analyze the causes of this failure and put forward our own insights to guide us on how to avoid similar problems in the future entrepreneurial process. (Case 105) I want colleges to provide relevant help so I can calmly analyze the causes of failure. This way, I can learn from my failures and grow stronger. (Case 132) What we need most is the sorting of the entrepreneurial process. (Case 112)	Entrepreneurial replication and failure attribution

In the startup stage, we need an entrepreneurial platform for communication and promotion. The communication platform allows us to better communicate with entrepreneurs or entrepreneurial teams. The promotion platform is to promote customers or provide suggestions through schools. (Case 16) I hope the utilization rate of relevant platforms increases, relevant venture capital support provided, and opportunities to communicate with successful entrepreneurial teams increase. (Case 38)	Entrepreneurship platform

We need the college to provide certain talent support or recommendation. (Case 49) We recruit professional teams with good knowledge background and practical experience, which are responsible for the product, technology, operation, and market. I believe a professional team will push our whole project to a higher level. (Case 44) I think we need more entrepreneurial team members. (Case 5)	Entrepreneurial team

Therefore, the policy support I need most is that the government or the school provides more preferential policies for college students. (Case 79) The support for entrepreneurship policy is most needed. (Case 73) Policies such as rent, store, and endowment insurance payments. (Case 84)	Entrepreneurial policies

What we need most now is family support. Parents always think that we should pay more attention to learning, and their knowledge of entrepreneurship is very limited. (Case 138) We need support from family the most, including money, ideas, etc. (Case 89)	Family support

Give us a free office to help us restart entrepreneurship. (Case 116) Give me some policy help and resources to help start my second business. (Case 124) Encourage re-entrepreneurship and offer a series of entrepreneurial help initiatives. (Case 107)	Re-entrepreneurship support

We need guidance on entrepreneurial projects. (Case 61) We need entrepreneurship mentor guidance, including guidance on finance, management, strategy, business model, etc. (Case 50) We need professional mentors from industry with practical experience to give us some direction in the biomedical industry to help break through the technical bottleneck. (Case 45)	Entrepreneurial guidance

We need financing, because it’s important. We are all grassroots entrepreneurs, without any accumulation of funds. Some time ago, we had a serious funding gap. We went to find some investors, sold 10% of the stock equity, and then we had one hundred thousand yuan (RMB). We were reluctant, but there was no other way. We have to rely on this money to survive. (Case 21) We need some funding support. Now our project is in the wintering period. It is most urgent to get through our profit model and establish an effective cash return. (Case 44) Financial support is most needed to help our company develop rapidly and attract more employees. (Case 92)	Financial support

At present, we need knowledge and technological support. (Case 24) I think what I need most is economic and technical support and experience accumulation. (Case 41) The biggest support needed for entrepreneurship is technical support. Only by solving technical problems can the company continue to develop. (Case 29)	Technical support

I don’t want to start a business again, and I didn’t pay attention to employment information, so I need someone to give me some occupational guidance afterward. (Case 110) At this time, I need occupational guidance, including curriculum vitae guide and job search assistance. (Case 117) Now that I have failed at starting a business, I’m ready to work for someone else. At present, I also need an employment guide to give me some information about career choices in the future and help me plan. (Case 122)	Occupational guidance

We need more brand-level planning to help us create a brand. (Case 29) We need the college to help promote our brand. (Case 28) Social awareness is our goal now. (Case 39)	Enterprise brand optimization

Schools should give us control over time. (Case 57) Academic pressure is high, and there is a time conflict. So, we need some time support. (Case 47)	Time support

For the students who fail to start their own business, schools should provide professional psychologists and entrepreneurial tutors for psychological counseling. I need this too. (Case 109) We need more psychological counseling to get out of the shadow of failure as soon as possible. (Case 118) At that time, when I was in a bad mood, I took the initiative to find a psychological counselor and asked her to help me out. (Case 136)	Psychological counseling

We need the recognition of our entrepreneurial team and the recognition of credit for entrepreneurial courses. (Case 33) At present, I need a more flexible “credit transfer system” policy. (Case 46) I hope that entrepreneurial experience can be counted as a course, and I can get course scores. (Case 9)	Credit transfer

At this stage, if our college can adopt our product, we will achieve rapid development. (Case 89) After discussion, our company cooperated with the logistics group of our college. It’s a great platform; the team has expanded and the turnover has increased. The company is running smoothly, and the school needs to continue to support the cooperation and expand new projects. (Case 68) We need funds and support from campus market channels. (Case 77)	Business support

The entrepreneurial atmosphere in our school is not strong. We need more training, so that we can gain more knowledge. (Case 7) Give our team a space suitable for self-development and provide relevant academic training support. (Case 45) I hope to get tax-, law-, and business-related learning and training. (Case 34)	Entrepreneurship training

I think what the studio needs most is social resources, such as various exhibitions and fairs, so that our studio can be more widely promoted. (Case 60) I hoped that there would be special guides to help us connect with the government and make useful policies and measures for us. (Case 64) Now we have a certain strength to do something. Moreover, we have our own base outside the school, so after graduation I can connect with the society and absorb social resources with the help of my college. (Case 7)	Resource connections

#### Entrepreneurship Competitions

A concept that originated in American universities in the 1980s, this can provide a valuable way to cultivate students’ entrepreneurial abilities and spirit. Studies have shown that entrepreneurship competition has a positive impact on entrepreneurial intention (Yu, 2013). Our interview data showed that entrepreneurship competition was an important support need for college student entrepreneurs: “I hope to participate in a business planning competition as soon as possible so that I can judge whether my idea is feasible and determine my next action plan” (Case 20).

#### Office Space

A place to operate is the most basic condition for entrepreneurial activities and startup development. A good workspace can mitigate the stresses of entrepreneurial activities. The ability to secure office space has become one of the most important factors affecting the success of college students, as the interviews revealed: “At present, what we need most is not only the assistance of professional mentors from the entrepreneurship college but also an office space suitable for our team” (Case 45).

#### Entrepreneurial Climate

This represents people’s value judgments and regional societal norms about entrepreneurship. Research has shown that the entrepreneurial climate has an important influence on the entrepreneurial decision-making and behaviors of college students (Huyghe and Knockaert, 2015). Our interview data confirmed this: “However, I think the atmosphere at the beginning is very important, and it has a great influence on people’s ideological understanding in the startup stage” (Case 12). We believe that a positive entrepreneurial climate is an important support need for college student entrepreneurs.

#### Entrepreneurial Replication and Failure Attribution

Our interviewees reported needing help with determining why their entrepreneurial efforts did not succeed: “I want colleges to provide relevant help so I can calmly analyze the causes of failure. This way, I can learn from my failures and grow stronger” (Case 132). The responses showed that entrepreneurial replication and failure attribution are also important support needs for college student entrepreneurs. Relevant studies show that reasonable attribution of entrepreneurial failure is an important way to help failed entrepreneurs out of the entrepreneurial failure dilemma and restart entrepreneurship learning to accumulate experience for the next startup or become outstanding staff for other entrepreneurs (Hisrich and Cahill, 1995).

#### Entrepreneurial Platform

This is an aggregate of entrepreneurial resources offered by institutions such as universities or governments. These resources play an essential role in promoting entrepreneurial activities. The academic community generally believes that the broad definition of entrepreneurship platforms includes the entrepreneurship base, entrepreneurship policies, entrepreneurship teachers, and other resources. In the narrow sense, the entrepreneurial platform usually refers to an entrepreneurial platform-based entity, and that is the sense referred to herein, which is why we broke down some of the specific components of the platform (e.g., office space and entrepreneurship policies). Our interview data showed that college entrepreneurs highly value entrepreneurial platforms: “In the startup stage, we need an entrepreneurial platform for communication and promotion. The communication platform allows us to better communicate with entrepreneurs or entrepreneurial teams. The promotion platform is to promote customers or provide suggestions through schools” (Case 16).

#### Entrepreneurial Team

Research has pointed out that the entrepreneurial team is one of the key elements for the success of a business (Clarysse and Moray, 2004). Knowing how to allocate tasks among entrepreneurial teams and continuously maintain work efficiency is important for entrepreneurs. “We recruit professional teams with good knowledge background and practical experience, which are responsible for the product, technology, operation, and market. I believe a professional team will push our whole project to a higher level” (Case 44). From the interviews, we learned that the entrepreneurial team is also an important support need for college student entrepreneurs.

#### Entrepreneurship Policies

Studies have shown that entrepreneurship policies have a significant effect on the formation of entrepreneurial intention, entrepreneurial decisions, and entrepreneurial activities (Lundström and Stevenson, 2005). This was confirmed by our interview data: “Policies such as rent, store, and endowment insurance payments” (Case 84).

#### Family Support

This refers to positive evaluations from and the approval and sometimes participation of entrepreneurs’ family members in their businesses. Research shows that family support plays an important role in entrepreneurial entry, continuance, and even exit, and this is also true for college student entrepreneurs (Song and Wang, 2019). Our interview data also confirmed this: “What we need most now is family support. Parents always think that we should pay more attention to learning, and their knowledge of entrepreneurship is very limited” (Case 138).

#### Re-entrepreneurship Support

This refers to support (including active investment) given by colleges and related organizations to college students who failed in their first attempt to start a business. Our interview data show that college students who failed at starting a business wanted the college to provide re-entrepreneurship support: “Encourage re-entrepreneurship and offer a series of entrepreneurial help initiatives” (Case 107).

#### Entrepreneurship Guidance

This describes the ways in which colleges, governments, and other institutions provide advice, suggestions, and direction to college entrepreneurs. Our interviews revealed that college students crave this support: “We need guidance on entrepreneurial projects” (Case 61).

#### Financial Support

Our interview data confirmed that the entrepreneurial college students considered financial support essential. “We need some funding support. Now our project is in the wintering period. It is most urgent to get through our profit model and establish an effective cash return” (Case 44). Financial support is one of the most-needed forms of support for college student entrepreneurs, and it is the most basic condition to ensure the smooth development of entrepreneurial activities (Zahra, 1993).

#### Technical Support

As the interview data show, technical support is important for college student entrepreneurs: “I think what I need most is economic and technical support and experience accumulation” (Case 41). “Technical support” refers to colleges providing technology services in the development of the core technologies needed by college entrepreneurs.

#### Occupational Guidance

This refers to a series of practical behaviors, such as employment consultation and skills training, adopted by the university, government, or other organizations to improve the competitiveness of employment when college students fail in their attempts to start businesses. As the interview data show, the students considered occupational guidance to be an important support requirement for college entrepreneurs: after a failed entrepreneurial attempt, one subject said, “Now that I have failed at starting a business, I’m ready to work for someone else. At present, I also need an employment guide to give me some information about career choices in the future and help me plan” (Case 122).

#### Enterprise Brand Optimization

This means employing all available resources to attract and engage more potential customers, and the subjects interviewed believed that college should provide more resources to help them package and promote their companies or products in a reasonable way to build a good reputation: “We need more brand-level planning to help us create a brand” (Case 29).

#### Time Support

In this context, “time support” refers to accommodative measures by the colleges to help entrepreneurial students reasonably arrange their time so they can devote enough time and energy to not just their academic tasks but also their startup ventures: “Academic pressure is high, and there is a time conflict. So, we need some time support” (Case 47).

#### Psychological Counseling

When college students’ entrepreneurial endeavors are not successful, they may feel overwhelmed by the sense of failure. Many of the subjects expressed a desire for the colleges to provide counselors who would help them deal with their feelings positively: “We need more psychological counseling to get out of the shadow of failure as soon as possible” (Case 118).

#### Credit Transfer

This refers to colleges acknowledging the valuable learning experiences in the students’ entrepreneurial activities by granting them academic credits for their efforts. The interview data showed that the subjects consider this important: “I hope that entrepreneurial experience can be counted as a course, and I can get course scores” (Case 9).

#### Business Support

This refers to the university or government giving purchase priority to products or services offered by college student entrepreneurs, which can directly help college student entrepreneurs increase business income and promote the development of startup enterprises: “After discussion, our company cooperated with the logistics group of our college. It’s a great platform; the team has expanded and the turnover has increased. The company is running smoothly, and the school needs to continue to support the cooperation and expand new projects” (Case 68).

#### Entrepreneurship Training

This refers to specialized training on topics specifically related to entrepreneurship, either as coursework or tutoring, according to the specific needs of college student entrepreneurs, in contrast to general entrepreneurial guidance. The subjects identified such training (or the lack of it) as having a significant effect on their entrepreneurial activities: “The entrepreneurial atmosphere in our school is not strong. We need more training, so that we can gain more knowledge” (Case 7).

#### Resource Connections

This describes the intermediary role of universities, governments, and other organizations in helping college student entrepreneurs expand their professional networks and form connections with external resources. The interviews showed that the subjects not only wanted but expected this type of support: “I hoped that there would be special guides to help us connect with the government and make useful policies and measures for us” (Case 64).

### Evaluation of the Importance of Support Needs

We conducted a frequency analysis of all the support needs by type and found that they varied in importance. The 10 most-desired forms of support were financial support, psychological counseling, entrepreneurial guidance, entrepreneurial replication and failure attribution, entrepreneurial policies, resource connections, occupational guidance, entrepreneurial team, technical support and Re-entrepreneurship support (see Table 3). Further analysis of the frequency of expressed interest in specific forms of support suggested that the core support needs (>5%) of college student entrepreneurs were financial support, psychological counseling, entrepreneurial guidance, entrepreneurial replication and failure attribution, entrepreneurial policies and resource connections. The secondary support needs (>2%) were occupational guidance, entrepreneurial team, technical support, re-entrepreneurship support, office space, and entrepreneurship platforms. Less frequently mentioned but still common enough to be considered marginal support needs (<2%) were (positive) entrepreneurial climate, entrepreneurship training, business support, entrepreneurship competitions, enterprise brand optimization, credit transfer, family support, and time support.

**TABLE 3:** Overall ranking of support needs of college student entrepreneurs.

Rank	Support needs	Frequency	Percentage
1	Financial support	45	21.03%
2	Psychological counseling	27	12.62%
3	Entrepreneurial guidance	25	11.68%
4	Entrepreneurial replication and failure attribution	23	10.75%
5	Entrepreneurial policies	15	7.01%
6	Resource connections	11	5.14%
7	Occupational guidance	9	4.21%
8	Entrepreneurial team	8	3.74%
9	Technical support	8	3.74%
10	Re-entrepreneurship support	7	3.27%
11	Office space	6	2.80%
12	Entrepreneurship platform	5	2.34%
13	Entrepreneurial climate	4	1.87%
14	Entrepreneurship training	4	1.87%
15	Business support	4	1.87%
16	Entrepreneurship competitions	3	1.40%
17	Enterprise brand optimization	3	1.40%
18	Credit transfer	3	1.40%
19	Family support	2	0.93%
20	Time support	2	0.93%

### Distribution of Support Needs Across the Entrepreneurial Stages

For this study, we broke the entrepreneurial process down into four stages: preparation, startup, maturity, and failure. To investigate the students’ ranking of the importance of the support needs during the different entrepreneurial stages, we conducted further frequency analysis on the distribution (Parker-Rhodes and Joyce, 1956). The results are shown in Table 4. As the table shows, no single support need was ranked above all others for all four stages. Seven of the support needs (i.e., financial support, entrepreneurial guidance, entrepreneurial policies, entrepreneurial team, technical support, entrepreneurship training, and credit transfer) were associated with three of the four entrepreneurial stages. Four needs (i.e., resource connections, office space, entrepreneurship platform, and time support) were associated with two stages. Nine needs (i.e., psychological counseling, entrepreneurial replication and failure attribution, occupational guidance, Re-entrepreneurship support, entrepreneurial climate, business support, entrepreneurship competitions, enterprise brand optimization, and family support) were associated with only one stage. Considering the distribution of the students’ ranking of support needs across the four stages, we found the following.

**TABLE 4:** Ranking of support needs of college student entrepreneurs by stage.

Support needs	Preparation stage	Startup stage	Maturity stage	Failure stage
Financial support	16	25	4	–
Psychological counseling	–	–	–	27
Entrepreneurial guidance	10	14	1	–
Entrepreneurial replication and failure attribution	–	–	–	23
Entrepreneurial policies	8	6	1	–
Resource connections	–	8	3	–
Occupational guidance	–	–	–	9
Entrepreneurial team	4	3	1	–
Technical support	2	4	2	–
Re-entrepreneurship support	–	–	–	7
Office space	1	5	–	–
Entrepreneurship platform	1	4	–	–
Entrepreneurial climate	4	–	–	–
Entrepreneurship training	1	2	1	–
Business support	–	4	–	–
Entrepreneurship competitions	3	–	–	–
Enterprise brand optimization	–	3	–	–
Credit transfer	1	1	1	–
Family support	–	2	–	–
Time support	1	1	–	–

#### Preparation Stage

This stage was associated with 12 support needs overall: three core support needs (financial support, entrepreneurial guidance, and entrepreneurial policies), four secondary support needs (entrepreneurial team, technical support, office space, and entrepreneurship platform), and five marginal support needs (entrepreneurial climate, entrepreneurship training, entrepreneurship competitions, credit transfer, and time support). For the preparation stage, the subjects’ mentions of their support needs, from most-frequent to least-frequent, were as follows: financial support, entrepreneurial guidance, entrepreneurial policies, entrepreneurial team, entrepreneurial climate, entrepreneurship competitions, technical support, office space, entrepreneurship platform, entrepreneurship training, credit transfer, and time support.

#### Startup Stage

This stage was associated with 14 support needs overall: four core support needs (financial support, entrepreneurial guidance, entrepreneurial policies, and resource connections), four secondary support needs (entrepreneurial team, technical support, office space, entrepreneurship platform), and six marginal support needs (entrepreneurship training, business support, enterprise brand optimization, credit transfer, family support, and time support). For the startup stage, the subjects’ mentions of their support needs, from most-frequent to least-frequent, were as follows: financial support, entrepreneurial guidance, resource connections, entrepreneurial policies, office space, technical support, entrepreneurship platform, business support, entrepreneurial team, enterprise brand optimization, entrepreneurship training, family support, credit transfer, and time support.

#### Maturity Stage

This stage was associated with 8 types of support needs overall: four core support needs (financial support, entrepreneurial guidance, entrepreneurial policies, and resource connections), two secondary support needs (entrepreneurial team and technical support), and two marginal support needs (entrepreneurship training and credit transfer). For the maturity stage, the subjects’ mentions of their support needs, from most-frequent to least-frequent, were as follows: financial support, resource connections, technical support, entrepreneurial guidance, entrepreneurial policies, entrepreneurial team, entrepreneurship training, and credit transfer.

#### Failure Stage

This stage was associated with only 4 types of support needs overall: two core support needs (psychological counseling and entrepreneurial replication and failure attribution) and two secondary support needs (occupational guidance and re-entrepreneurship support). No marginal support needs were mentioned. For the failure stage of entrepreneurship, the subjects’ mentions of their support needs, from most-frequent to least-frequent, were as follows: psychological counseling, entrepreneurial replication and failure attribution, occupational guidance, and re-entrepreneurship support. We must highlight that, as an important form of psychotherapy, psychological counseling plays an important role in helping college students get out of the shadow of entrepreneurial failure. However, as far as we know, the colleges and other relevant stakeholders were not paying enough attention to this.

## Discussion

On the basis of information gleaned from the literature review, this study identified the main support needs of college student entrepreneurs, evaluated the importance of different support needs, and explored the distribution tendencies of support needs across different entrepreneurial stages using the standard case study method. The findings showed that the college student entrepreneurs interviewed were concerned with at least 20 support needs, which is partly similar to the findings of Van Weele et al. (2016).

We also evaluated the importance of the different support needs according to college student entrepreneurs, categorizing them into core support needs, secondary support needs, and marginal support needs. Unlike general entrepreneurs who focus on business models and market access, the college student entrepreneurs in the study were more focused on financial support, psychological counseling, and entrepreneurial guidance. Of those three, they considered financial support to be the most important; this finding is consistent with research on entrepreneurs in general (Van Weele et al., 2016). Psychological counseling ranked second, indicating that college student entrepreneurs were both open to and in need of guidance and advice on dealing with their emotions as they related to their entrepreneurial endeavors—especially when those endeavors were unsuccessful. This finding differs from those of some studies on support needs but is consistent with Yamakawa and Cardon’s (2015) finding on the causal ascriptions and perceived learning from entrepreneurial failure. Our findings seem reasonable given that the average psychological maturity and confidence gained through experience by college students would be lower than that of entrepreneurs in general. More mature and experienced entrepreneurs would probably be more resilient when their entrepreneurial efforts did not succeed; in contrast, college students would be more likely to need the buffer of psychological counseling to help them through the maelstrom of feelings in the wake of an entrepreneurial failure. Another possible reason why the subjects may have placed such a high value on psychological counseling is that the emotional aspects of entrepreneurship are often neglected in entrepreneurial education and training; coursework may be better at encouraging entrepreneurial students to expect success than at preparing them to see failure as a useful lesson on the way to eventual success. Entrepreneurial guidance ranked third, most likely because the students’ relative youth would mean a relative lack of resources and experience. In line with others’ findings (Lockyer and George, 2012; Vandor et al., 2012), we found that financial support, entrepreneurial guidance, entrepreneurial policies, and entrepreneurial connections were support needs commonly cited among entrepreneurs; not surprisingly, credit exchange and time support were support needs cited only by college student entrepreneurs.

We also considered the time dimension in this study and found that the subjects ranked the support needs differently for each of the four entrepreneurial stages, which is consistent with the findings of Vandor et al. (2012). We also found that the college student entrepreneurs needed the most support during the startup stage of entrepreneurship, and this is also consistent with the research (Ge et al., 2016). Our examination of the cross-stage distribution of support needs showed some similarity between the preparation stage and the startup stage and markedly different results for the failure stage compared with the other three stages. This is partly similar to the findings of other scholars on entrepreneurial stage distribution (Clarysse and Moray, 2004).

## Conclusion

This study identified 20 types of support needs for college student entrepreneurs: financial support, psychological counseling, entrepreneurial guidance, entrepreneurial replication and failure attribution, entrepreneurial policies, resource connections, occupational guidance, entrepreneurial team, technical support, re-entrepreneurship support, office space, entrepreneurship platform, entrepreneurial climate, entrepreneurship training, business support, entrepreneurship competitions, enterprise brand optimization, credit transfer, family support, and time support. We evaluated the relative importance of the different support needs, categorized them into core support needs, secondary support needs, and marginal support needs. Among the 20 types overall (that is, not by entrepreneurial process stage), there were six core support needs (financial support, psychological counseling, entrepreneurial guidance, entrepreneurial replication and failure attribution, entrepreneurial policies, and resource connections), six secondary support needs (occupational guidance, entrepreneurial team, technical support, re-entrepreneurship support, office space, and entrepreneurship platform), and eight marginal support needs (entrepreneurial climate, entrepreneurship training, business support, entrepreneurship competitions, enterprise brand optimization, credit transfer, family support, and time support).

This study also explored the distribution of the support needs across the four entrepreneurial stages and found that the entrepreneurial college students ranked the needs differently for each. We identified 12 critical support needs for the preparation stage, 14 types for the startup stage, 8 types for the maturity stage, and 4 types for the failure stage. Further, none of the support needs appeared in the four entrepreneurial stages at the same time. However, 7 types of support needs appeared in three of the four stages; 4 types appeared in two of the four stages; and 9 types appeared in only one stage. We also found that 4 types of support needs appeared only in the failure stage.

The conclusions of this study should help the academic community to better understand the support needs of college student entrepreneurs and develop policies and practices to provide that support. On the basis of our research findings, we recommend that the government, colleges, and other stakeholders consider the following to provide better support for college student entrepreneurs and promote their success. First, relevant stakeholders should provide immediate financial support and effective entrepreneurial guidance, as these are the basic resources college students require to start their own businesses. Second, entrepreneurship-positive institutional policies and practices need to be established to make the entrepreneurial process sustainable. Research has shown that positive entrepreneurship policies are among the core support needs of college student entrepreneurs. Empirically proven entrepreneurial policies to promote the success and sustainability of college student entrepreneurs need to be implemented. Third, our research showed that the college students wanted and needed ready access to psychological counseling, but the colleges and other relevant stakeholders were not paying enough attention to this. The “hard side” of entrepreneurship development (e.g., financial support, office space, entrepreneurship platform) needs to be complemented by the “soft side” (e.g., emotional and psychological counseling) to prepare college student entrepreneurs for the real-world ups and downs of entrepreneurship.

This study had some limitations. First, although we collected qualitative research data from 138 college student entrepreneurs through primary and secondary methods, the sample size was still small relative to the numbers of college student entrepreneurs. Second, the data and samples were all from Chinese college student entrepreneurs. Although the subjects were from 14 different provinces, we did not have subjects from every province. None of the subjects were Chinese nationals attending colleges in other countries, nor were any of them non-Chinese students attending colleges in China or in other countries. In the future, the study should be replicated with a larger sampling of students, a broader geographical distribution, and non-Chinese students and colleges. Third, in assessing the importance of the support needs, we only used frequency statistics as a measure. Although this method has been used many times, we still cannot deny its disadvantages. Future studies should consider alternative and additional measurement methods. Finally, from an overarching perspective, this study belongs to the category of exploratory research based on qualitative data. In the future, more rigorous quantitative research should be conducted to deepen the understanding of the support needs of college student entrepreneurs.

## Data Availability Statement

The raw data supporting the conclusions of this article will be made available by the authors, without undue reservation, to any qualified researcher.

## Ethics Statement

The studies involving human participants were reviewed and approved by Institutional Review Board of the Institute of China Innovation and Entrepreneurship Education at Wenzhou Medical University. Written informed consent for participation was not required for this study in accordance with the national legislation and the institutional requirements. Written informed consent was obtained from the individual(s) for the publication of any potentially identifiable images or data included in this article.

## Author Contributions

PW contributed to research design, data analysis, and manuscript writing. YH contributed to the research design and provided quality assurance of the research. Both authors contributed to the article and approved the submitted version.

## Conflict of Interest

The authors declare that the research was conducted in the absence of any commercial or financial relationships that could be construed as a potential conflict of interest.
